# A stable isotope dual-labelling approach to detect multiple insemination in un-irradiated and irradiated *Anopheles arabiensis *mosquitoes

**DOI:** 10.1186/1756-3305-1-9

**Published:** 2008-04-10

**Authors:** Michelle EH Helinski, Rebecca C Hood, Bart GJ Knols

**Affiliations:** 1 International Atomic Energy Agency (IAEA), Agency's Laboratories Seibersdorf, A-2444 Seibersdorf, Austria; 2 Laboratory of Entomology, Wageningen University and Research Centre. P.O. Box 8031, 6700 EH Wageningen, The Netherlands

## Abstract

**Background:**

In the context of a Sterile Insect Technique programme, the occurrence of multiple insemination in the malaria mosquito *Anopheles arabiensis *Patton was studied using a novel labelling system with the stable isotopes ^15^N and ^13^C. The incidence of multiple insemination in the absence of radiation, and when males were irradiated in the pupal stage and competed against un-irradiated males were assessed. Males used in the experiments were labelled with either ^15^N or ^13^C and the label was applied to the larval rearing water. Males with either label and virgin females were caged at a 1:1:1 ratio. Males used in the radiation treatments were irradiated in the pupal stage with a partially or fully-sterilizing dose of 70 or 120 Gy, respectively. After mating, females were dissected and inseminated spermathecae analysed using mass spectrometry.

**Results:**

The data indicate that about 25% of inseminated females had been inseminated multiply. The presence of irradiated males in the experiments did not affect the incidence of multiple insemination. In line with previous research, irradiated males were generally less competitive than un-irradiated males.

**Conclusion:**

The implications of these findings for the Sterile Insect Technique are discussed, and further experiments recommended. The dual-labelling system used to determine paternity gave good results for ^13^C, however, for ^15^N it is recommended to increase the amount of label in future studies.

## Background

Polyandry, insemination of a female by more than one male, is a common phenomenon in many insects [1], but malaria mosquitoes (*Anopheles *spp.) are generally believed to be largely monandrous (i.e. insemination of a female by a single male) under natural conditions [2]. However, some level of polyandry (named "multiple insemination" for the rest of this paper) has been observed both in laboratory and field studies. The occurrence of multiple insemination has been assessed in field populations using molecular markers [3,4]. Multiple insemination occured in low frequencies (i.e. 2.5%) in *Anopheles gambiae *Giles [4] and similar results were observed in *An. freeborni *Aitken [3]. In laboratory cages much higher frequencies of multiple insemination can be observed (i.e. using eye-colour mutants [5-9]). In *An. gambiae s.s*., Gomulski [7] reported 12% multiple insemination after a mating period of 6 days using mosquitoes at a ratio of 1:1:2 (i.e. male: male: female), while 11–48% of multiple insemination was observed in another study (i.e. depending on the timing of remating, after or within 24 hrs of the first insemination, using a 1:1:1 ratio [9]). In *An. culicifacies *Giles, multiple mating was assessed for various densities of mosquitoes at a ratio of 1:1:2 and a positive correlation between insect density and multiple insemination was observed [6].

Genetic control programmes including the Sterile Insect Technique (SIT) rely on the ability of released males to successfully inseminate wild females and introduce their genes into the next generation. In principal, polyandry is not considered to be a drawback in genetic control programmes as long as the sperm of released males is able to compete with sperm from wild males after insemination [10]. Under these conditions polygamy is advantageous, as it would result in the re-mating of immigrating females from neighbouring untreated sites.

In SIT programmes, irradiation is routinely used to induce sterility. In previous work it was determined that *An. arabiensis *Patton males irradiated as pupae produced fewer and smaller sperm compared to un-irradiated males (M. Helinski, unpublished results). Even though it is not known if small sperm contribute to fertilization in *Anophele*s, the observation that females select for larger sperm in their spermathecae [11], coupled to results from studies performed in *Drosophila *where small sperm are not used for fertilization [12], might indicate that they are of little importance for fertilization. The implications of these findings on mating behaviour of anophelines is not known; however, in the Mediterranean fruit fly *Ceratitis capitata *Wiedemann it was observed that sterile males transferred fewer sperm than wild type males and females mated to irradiated males were more likely to re-mate compared to females mated to un-irradiated males [13]. The aim of the present study was to assess the occurrence of multiple insemination events in a laboratory colony of *An. arabiensis*, and to determine the influence of irradiation at the pupal stage with a partially or fully-sterilizing dose on the occurrence of these events during competition experiments.

Multiple insemination events were determined using a dual-labelling system of stable isotopes. Stable isotopes are naturally occurring in the environment, are not radioactive, and react in general chemically identical to the more common isotope. These attributes make them effective non-invasive markers in biological systems [14]. In previous work it was established that the stable isotopes ^13^C and ^15^N could be used individually as semen labels [15,16]. The labels were applied in the larval rearing water, and labelled males transferred sufficient label to permit the positive identification of the label in spermathecae after mating [15,16]. Because both isotopes can be simultaneously analysed in a sample using an elemental analyser linked to an isotope ratio mass spectrometer, a device that separates ions of the element of interest on the basis of their differing mass/charge ratio (m/z) (see Hood-Nowotny and Knols [14] for details), in this study the use of both labels to determine multiple insemination was studied.

## Methods

### Mosquitoes and labelling

The mosquito strain used in all experiments was the Dongola strain of *An. arabiensis *Patton (available from MR4, CDC Atlanta, USA) colonised in 2004 from specimens collected near Dongola, Northern State, Sudan. This strain has been maintained in the laboratory for approximately sixty generations. Insects were reared according to the methods described in Helinski et al. [15]. Briefly, five hundred L1 stage larvae were counted and placed in a tray (30 × 40 cm) in 1.5 L of deionized water, and the label was added to the larval water on the same day as the L1 larvae were introduced. Larvae were fed a fixed diet of fish food (approx. 0.25 mg/larva/day, AquariCare Koi Floating Blend, USA); adult mosquitoes were continuously supplied with a 6% sucrose solution [w/v]. Mosquitoes were labelled in the larval stage with ^15^N-glycine or ^13^C-glucose following procedures described in Helinski et al. [15,16]. For both isotopes, the target enrichment in the mosquito was 5 atom%. For ^15^N this meant that 5 atom% of ^15^N was added (i.e. 5% of all the nitrogen in the diet was ^15^N), while for ^13^C 20 atom% was added due to losses resulting from turnover and respiration [15,16].

### Experimental procedures

Males from the labelled trays were separated from females and maintained as virgins before mating experiments took place. All females used as mates came from trays unlabelled with isotopes and females were sexed < 18 hrs after emergence to assure virginity.

Competition experiments were performed where irradiated and un-irradiated groups of males competed for females in a 1:1:1 ratio. For each ratio 34–40 insects were used. The experiments were performed in standard rearing cages (30 × 30 × 30 cm) and the mosquitoes were left together for 4 nights. Age of the mosquitoes at the start of the experiment was between 3–5 days. The two groups of males were labelled with either ^15^N or ^13^C. Males were irradiated in the pupal stage following procedures as described in Helinski et al. [17], with a partially or fully sterilizing dose of 70 or 120 Gy, respectively. To determine the degree of multiple mating in the absence of radiation, un-irradiated males labelled with either label competed for females (i.e. control treatment). For each dose, 2 sets of experiments were performed and each set consisted of three treatments:

1) Control: ^15^N un-irradiated males : ^13^C un-irradiated males : virgin females,

2) ^15^N irradiated males : ^13^C un-irradiated males : virgin females,

3) ^15^N un-irradiated males : ^13^C irradiated males : virgin females. Treatments 2 and 3 were the same except that the isotope label was alternated between groups of males.

Males used in each set of experiments originated from the same batch of eggs, and were reared under standardized larval conditions (i.e. density, food, and water depth) in their respective labelled trays. For each set of experiments, it was attempted to use males of exactly the same age, however this was not always feasible and in experiments three and four males differing in age by 1 day were used.

After the mating period, females were dissected for assessment of insemination and for each treatment inseminated spermathecae (N = 17–20) were prepared for analysis in the mass spectrometer [15,16]. For each set of experiments, spermathecae from virgin females (N = 5) were included. Samples were spiked to attain sufficient nitrogen or carbon to be above the detection limit of the isotope ratio mass spectrometer setup [15,16], and the spike used for these samples consisted of 20 μl of a solution containing 20 μg N and 25 μg C.

### Data analysis and interpretation

These analysis and interpretation were similar to Helinski et al. [15,16], and the spiked delta values were used for data analysis. The δ values reported are referenced to the international standards for nitrogen (i.e. AIR) and carbon (i.e. Vienna Pee Dee Belemite). Samples were analysed at the stable isotope facility at UC Davis (Davis, USA). To determine if a spermatheca was inseminated by a ^15^N or a ^13^C labelled male, threshold values based on 2 (for ^15^N) or 3 (for ^13^C) standard deviations above the mean values observed for virgin females were used [15,16,18]. Mean values were based on all virgin samples across the experiments.

There was very little variation in mean δ^13^C‰ values for virgin samples (*M *= -25.86, s.d. 0.16), and the threshold value was set at 3 s.d. above the average value. The mean δ^15^N‰ values were more difficult to interpret; substantial variation was observed, and two δ^15^N‰ values of virgin females were removed from the dataset to normalise the data (i.e. -0.94 and -1.71). One high δ^15^N‰ was observed in a control (treatment 1) replicate (i.e. 15.88); this data point was included as a ^15^N positive sample throughout the sample analysis but removed from the analysis of the mean ± s.d. δ^15^N‰ for labelled samples. The standard deviation for mean virgin samples remained quite large (*M *= -4.08, s.d. 0.68), and a threshold value of 2 s.d. was used.

### Statistics

Prior to analyses, data were checked for normality. To determine competitiveness, the number of spermathecae inseminated by ^15^N or ^13^C labelled males (in the case of radiation studies these were categorized as irradiated versus un-irradiated; Table 1) were analysed using a replicated G-test of goodness of fit that allowed for the analysis of replicates within a treatment. If there was no heterogeneity between replicates of a treatment data were pooled (see Sokal and Rohlf [19] for more details). Even though in Table 1 the data are grouped separately per category (e.g. for ^15^N only spermathecae positive for ^15^N are grouped), for G-test analyses spermathecae in which both labels were present were added to obtain the total number of spermathecae inseminated by either group of labelled males. The proportion of multiple mating events between treatments (i.e. control and irradiation with 70 or 120 Gy) were analysed with General Linear Models (GLMs) and means were separated using Tukey's Honestly Significantly Different (HSD) or individual t-tests. Differences in insemination rate between the three treatments were analysed with GLMs. All two-sided tests were performed using the SPSS software version 14 (SPSS Inc., Chicago, USA).

**Table 1:** Results from the competition experiments using isotope labelled males (i.e. ^15^N or ^13^C) to determine insemination in spermathecae. Treatments were 1) ^15^N un-irradiated males : ^13^C un-irradiated males : females (i.e. control), 2) ^15^N irradiated males : ^13^C un-irradiated males : females, 3) ^15^N un-irradiated males : ^13^C irradiated males : females. Males were irradiated with 70 or 120 Gy in the pupal stage. For the control treatment 1, the proportion and the number (in between brackets) of spermathecae positive for ^15^N or ^13^C, are indicated. For the radiation treatments, spermathecae inseminated by irradiated or un-irradiated males are shown. Spermathecae positive for both isotopes are classified as inseminated by "Both". Those with amounts of isotopes below the detection threshold are classified as 'Neither". N is the total number of spermathecae analysed per row. Individual and pooled (when replicates could be grouped) G-test results for each competition experiment are given (see text for details) with significant differences at * p < 0.05 and ** p < 0.01. Average values for competition experiments (insemination by irradiated or un-irradiated males only) are only given when replicates could be grouped.

Control	Exp.	Treatment 1	% ^15^N (N)	% ^13^C (N)	% Both isotopes (N)	% Neither isotope (N)	N	G-test ^15^N vs ^13^C
	1	1	65 (13)	20 (4)	15 (3)	0 (0)	20	3.62
	2	1	60 (12)	10 (2)	30 (6)	0 (0)	20	3.95*
	3	1	25 (5)	60 (12)	10 (2)	5 (1)	20	2.38
	4	1	45 (9)	45 (9)	0 (0)	10 (2)	20	0
		average	n/a	n/a	14 ± 6	4 ± 2		n/a

70 Gy	Exp.	Treatments 2–3	% Irrad. (N)	% Un-irrad. (N)	% Both isotopes (N)	% Neither isotope (N)	N	G-tests 70 vs 0 Gy

	1	2 ^15^N	35 (7)	35 (7)	30 (6)	0 (0)	20	0
	1	3 ^13^C	15 (3)	65 (13)	20 (4)	0 (0)	20	4.30*
	3	2 ^15^N	15 (3)	65 (13)	0 (0)	20 (4)	20	6.74**
	3	3 ^13^C	35 (7)	55 (11)	10 (2)	0 (0)	20	0.73
		average	25 ± 6	55 ± 7	15 ± 6	5 ± 5		6.63*

120 Gy	Exp.	Treatments 2–3	% Irrad. (N)	% Un-irrad. (N)	% Both isotopes (N)	% Neither isotope (N)	N	G-tests 120 vs 0 Gy

	2	2 ^15^N	18 (3)	65 (11)	18 (3)	0 (0)	17	3.29
	2	3 ^13^C	42 (8)	53 (10)	5 (1)	0 (0)	19	0.20
	4	2 ^15^N	0 (0)	95 (19)	5 (1)	0 (0)	20	21.07**
	4	3 ^13^C	30 (6)	65 (13)	5 (1)	0 (0)	20	2.38
		average	n/a	n/a	8 ± 3	0		n/a

n/a: not applicable (replicates could not be grouped based on G-test results)

## Results

After 4 nights of mating, on average 92 ± 2% (± s.e.m.) of all females were inseminated and no significant differences were observed between treatments (i.e. treatments 1, 2 and 3; F_2,9 _= 0.42, p > 0.05). Irradiation, therefore, had no impact on insemination in these competition experiments.

**Figure 1:**
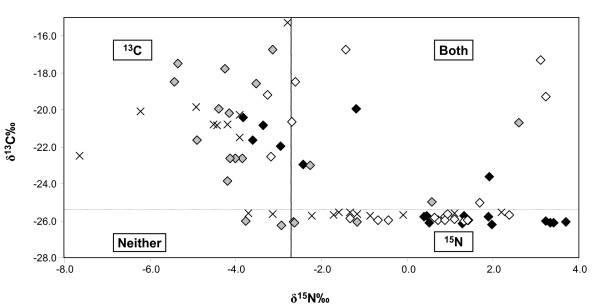
**δ^13^C‰ and δ^15^N‰ values of spermathecae from the control experiments, treatment 1**. The four symbols indicate samples from the 4 replicates. Threshold values were defined as 2–3 standard deviations (s.d.) above the mean value of virgin spermathecae samples, and are indicated for δ^15^N‰ (2 s.d. solid line) and δ^13^C‰ (3 s.d. broken line). Experimental samples could thus be defined as ^13^C labelled (top left quadrant), ^15^N labelled (bottom right quadrant), labelled with both isotopes (Both, top right quadrant) or no label was detected (Neither, bottom left quadrant).

The insemination of spermathecae by either or both groups of labelled males was determined based on the threshold values for δ^15^N‰ and δ^13^C‰. The classification of spermathecae into the different labelling groups is illustrated for the control, treatment 1 (Fig. 1). Even though all spermathecae were inseminated, in some samples the label was below the detection limit and these samples were classified as not having sufficient label (i.e. indicated as "Neither", Fig. 1 and Table 1). For all data combined, the mean delta values ± s.d. of samples classified as ^15^N labelled was 0.09 ± 2.01 (N = 124), and for unlabelled samples -4.26 ± 0.98 δ^15^N‰ (N = 112). For ^13^C, mean δ^13^C‰ values were -20.80 ± 2.00 (N = 129) for labelled and -25.86 ± 0.17 (N = 107) for unlabelled samples.

In the absence of radiation, cases of multiple mating were observed in three out of the four replicates for the control treatment 1, and on average 14 ± 6% of inseminated spermathecae were positive for both ^13^C and ^15^N (Table 1). The competitiveness of both groups of males was determined, however replicates differed statistically and data could not be grouped (G_3 _= 8.30, p < 0.05). The individual G-tests showed that the competitiveness of ^13^C or ^15^N labelled males was similar with the exception of replicate two where ^15^N labelled males inseminated significantly more females than ^13^C labelled males (Table 1).

When males were irradiated with 70 Gy and competed against un-irradiated males, replicates were statistically similar and data were grouped (G_3 _= 5.14, p > 0.05). Un-irradiated males had a higher competitiveness than irradiated males (Table 1), and on average 55 ± 7% of females were inseminated by un-irradiated males only compared to 25 ± 6% by irradiated males only (Table 1). Cases of multiple mating were observed in three out of four replicates; levels of multiple mating observed were comparable to treatment 1 and on average 15 ± 6% of the spermathecae were positive for both labels (Table 1).

For 120 Gy, the distributions of females inseminated by irradiated or un-irradiated males were not similar and replicates could not be pooled (G_3 _= 10.59, p < 0.01). In three out of four replicates males irradiated with 120 Gy inseminated fewer females than un-irradiated males, however only in the third replicate a significant result was observed (Table 1). In replicate 2 irradiated males inseminated similar numbers of females compared to un-irradiated males. Multiple mating occurred in all replicates and on average 8 ± 3% of the spermathecae were positive for ^15^N and ^13^C.

When data were combined, there were no significant differences in the proportion of multiple mating events for all treatments (F_2,9 _= 0.42, p > 0.05), thus the irradiation of one group of males with either dose had no impact on the proportion of multiple insemination compared to the control treatment. The alteration of the label for the irradiated males group did not have an impact on the proportion of multiple mating events, and similar results were observed for treatments 2 and 3 for both doses (t(6) = 0.43, p > 0.05).

## Discussion

Using an entirely new method of stable isotope labelling, the findings of this study show that multiple insemination occurs in a laboratory colony of *An. arabiensis*. On average 12 ± 3% of spermathecae were labelled with both ^15^N and ^13^C. It is assumed that undetected double mating events (e.g. ^13^C followed by a second ^13^C insemination) occurred to the same extent [7,9], thus the actual number of multiple insemination is about two-fold higher, and approximately 25% of all females were inseminated more than once. A higher frequency of multiple insemination was observed compared to results for *An. culicifacies *(i.e. 12–14% [6]), and *An. gambiae s.s*. (i.e. 12% [7]), however in those studies the proportion of females used was two-fold higher compared to this study. At a similar ratio of insects compared to this study (i.e. 1:1:1), a higher frequency of multiple insemination was observed in *An. gambiae s.s*. after 1 night of mating (i.e. 48% [9]).

Irradiation appeared to have no impact on the frequency of multiple insemination in this experimental design; for 70 Gy similar frequencies were reported compared to control treatments, while for 120 Gy lower frequencies were found but differences were not significant. Even though pupal irradiated males have fewer and smaller sperm in their testes [M. Helinski, unpublished results], in treatments where these males competed against un-irradiated males for females, no increase in multiple insemination was observed. Females inseminated by males irradiated in the pupal stage were thus not more likely to engage in another mating event with an un-irradiated male. This is in contrast to results for the Mediterranean fruit fly, where females inseminated by irradiated males were more likely to re-mate [13]. However, males were introduced sequentially (i.e. between 1–3 days after first mating) while in the present study males were introduced simultaneously. In addition, mating behaviour of both species is completely different (i.e. the Mediterranean fruit fly mating system is based on female-choice, while in *Anopheles *a female appears to have little choice in copulation partner upon entry in a swarm). However, for both species a signal from the sperm is thought to regulate female mating behaviour [9,20], and it thus seems that pupal irradiated *An. arabiensis *males transfer enough sperm to trigger this behaviour. As discussed, polyandry in anophelines would be advantageous for Sterile Insect Technique programmes on the premise that sperm of released males is equally competitive to wild males [10]. The competitiveness of irradiated sperm compared to un-irradiated sperm for fertilization of the eggs was not determined in this study, and only the incidence of multiple insemination was scored. In future studies it would be necessary to determine which sperm (i.e irradiated or un-irradiated) the female uses to fertilise her eggs.

The competitiveness of irradiated males was lower compared to un-irradiated males in the majority of replicates for 70 and 120 Gy. For 70 Gy, replicates could be pooled and 55 ± 7% of the females were inseminated by un-irradiated males only. In previous studies, hatch data of individual egg batches were used to determine paternity, and un-irradiated males accounted for 58 ± 5% and 75 ± 5% of the egg batches for 70 and 120 Gy experiments, respectively [21], thus results were comparable for 70 Gy. Hatch data of egg batches permits the identification of batches with intermediate hatch, which is assumed to be the result of a multiple mating event [21-24]. Data from small cage competition experiments at a 1:1 ratio observed 10% and 5% of the egg batches with intermediate hatch rate (i.e. between 20–60%) for 70 and 120 Gy, respectively [21], which is similar to the values observed in this study, (i.e. 12.5% and 8% for 70 and 120 Gy, respectively).

A review of the literature shows that different results on multiple insemination are observed between field and laboratory studies [4]. In the former, multiple insemination only occurred in very low frequencies as assessed using molecular markers [3,4], while in the latter a high proportion of multiple insemination can be found using mutant strains [6,7,9]. In the field, mating couples usually leave the swarm *in copula *[25], and after a successful mating females probably leave the swarm site. In laboratory cages, females are confined with males throughout the swarming period, usually in small cages (i.e. 30 cm square cages are common) at high densities. It is therefore likely that females are forced to fly into the swarm on several occasions due to disturbances in the cage which facilitates remating. Alternatively, it has been suggested that in small cages mating is interrupted (i.e. due to space restriction when flying *in copula*, or other males disturbing the couple) resulting in the incomplete transfer of semen making females more responsive to a subsequent mating [6]. This suggestion was based on results from a study in *An. culicifacies*, which found that when cage size was increased and insect density decreased, multiple insemination decreased [6]. From the above it is evident that multiple mating studies should be repeated in much larger cages (e.g. semi-field systems [26]) to exclude disturbances and space issues as the possible explanations for the observed results.

The dual-labelling approach with two different isotopes assumed equal mating ability of both groups of labelled males. In previous studies, the mating ability of ^13^C-glucose and ^15^N-glycine labelled males were similar to unlabelled males [15,16], and for ^13^C it was determined that the use of ^13^C-labelled glucose or unlabelled glucose had no impact on larval development and male longevity compared to a control with no glucose [15]. In three out of four replicates in the control treatment, ^15^N or ^13^C labelled males were equally competitive, while in the other replicate ^15^N males were just significantly more competitive (p = 0.047). In the irradiation experiments no specific pattern was observed to indicate that males of a particular label performed better compared to the other group (Table 1). For the different labelling treatments, different carrier compounds for the isotopes were used, i.e. glycine and glucose. Even though in our initial data comparable levels of larval survival were observed for larvae reared with ^13^C-glucose [15], in these experiments a reduced growth rate and survival in ^13^C trays was sometimes observed compared to unlabelled or ^15^N-glycine trays. In larvae of *Aedes aegypti *Wiedemann the addition of glucose at high concentrations was observed to slow development rates [27], and it is likely that such an effect occasionally took place after ^13^C-glucose was added. It should be noted that labelling with ^13^C-glucose is possible but adequate attention should be given to prevent a reduction in growth. Glycine did not belong to the class of essential amino-acids needed for development in *Aedes aegypti *L. larvae; however, in *Culex pipiens *L. glycine was essential to achieve normal growth rates [27]. No such data for anophelines are available, however the standard diet provided to the larvae in our insectary always resulted in normal growth and development; and it thus seems unlikely that ^13^C-glucose labelled males were deprived of glycine. In future experiments it might nevertheless be worthwhile to add unlabelled glycine to the ^13^C-glucose labelled trays and vice versa.

The threshold values used in this study classified some spermathecae as unlabelled even though they were visually inseminated; however, only about 3% of the spermathecae were in this category. A careful look at the data indicated that the majority of the difficulties in the data analysis were caused by the δ^15^N‰ values, and a greater variation in unlabelled samples was observed compared to the δ^13^C‰ values. Even though in previous work an enrichment of 5 atom% ^15^N was sufficient to distinguish inseminated spermathecae from uninseminated ones [16], in the present study we conclude that a higher enrichment would have resulted in a better spread of the data for labelled and unlabelled samples. Samples in this study were analysed at a different facility compared to previous work [16], and this could have accounted for the differences observed. It is recommended in future experiments to increase the enrichment of ^15^N labelled males, for instance double the amount of label could be used. This would only result in a marginal increase in costs (i.e. 5 US$ versus 2.5 US$ per larval tray) compared to ^13^C labelling (i.e. 25 US$ per larval tray). The impact of an increase of^15^N-glycine on larval development however, needs to be tested, as high amino-acid concentrations could be deleterious [27].

## Conclusion

In conclusion, the first time use of a dual-labelling system of stable isotopes in mating studies gave evidence for multiple insemination events in a laboratory strain of *An. arabiensis*. Further optimisation of the method is possible, and the use of a higher enrichment of ^15^N-labelled males is recommended for future experiments. Discrepancies between field and laboratory collected data indicate that these experiments should be repeated in larger (semi-field) cages to avoid artificially forcing females into repeated contact with males. In the context of genetic control studies with the Sterile Insect Technique, experiments were performed to determine the effects of radiation on the frequency of multiple mating. In small cages, the irradiation of males had no impact on the proportion of multiple insemination events when these males competed with un-irradiated males for mates. Further studies on the sequential introduction of mates and the determination of sperm use for fertilization are recommended.

## Authors' contributions

MEHH designed and performed the experiments, analysed the results and wrote the manuscript. RCH advised on stable isotope use, assisted with data analysis and drafting of the manuscript. BGJK supervised the work and helped to draft the manuscript. All authors read and approved the final manuscript.
